# Supplementary material for: A Critical View of the Application of the APEX Software (Aqueous Photochemistry of Environmentally-Occurring Xenobiotics) to Predict Photoreaction Kinetics in Surface Freshwaters
Source: Molecules. 2019 Dec 18;25(1):9. doi: 10.3390/molecules25010009 (PMC7017383; doi:10.3390/molecules25010009)
Supplement: Supplementary file 1 [file molecules-25-00009-s001.zip › 3.2.4_gcc-4.4.0.zip!/3.2.4_gcc-4.4.0/3.2.4_gcc-4.4.0/doc/octave/html/interpreter/Oct_002dFiles.html]

Oct-Files - Untitled


Next: Mex-Files,
Up: Dynamically Linked Functions


---

### A.1 Oct-Files

- Getting Started with Oct-Files- Matrices and Arrays in Oct-Files- Character Strings in Oct-Files- Cell Arrays in Oct-Files- Structures in Oct-Files- Sparse Matrices in Oct-Files- Accessing Global Variables in Oct-Files- Calling Octave Functions from Oct-Files- Calling External Code from Oct-Files- Allocating Local Memory in Oct-Files- Input Parameter Checking in Oct-Files- Exception and Error Handling in Oct-Files- Documentation and Test of Oct-Files
